# Steroid resistance in organizing pneumonia caused by pulmonary cryptococcosis

**DOI:** 10.1002/rcr2.556

**Published:** 2020-04-09

**Authors:** Motoko Nomura, Hiromitsu Ohta, Masahiro Hiruta, Fumiaki Kudo, Fumiyoshi Ohyanagi, Yasuhiro Yamaguchi

**Affiliations:** ^1^ Department of Respiratory Medicine Jichi Medical University Saitama Medical Center Saitama Japan; ^2^ Department of Pathology Jichi Medical University Saitama Medical Center Saitama Japan

**Keywords:** Cryptococcosis, cryptogenic organizing pneumonia, immunocompromised, transbronchial lung biopsy

## Abstract

Cryptogenic organizing pneumonia (COP) usually responds well to steroid therapy; however, recurrence is commonly observed when the steroid dose is tapered. A 74‐year‐old man suspected of having steroid‐resistant COP presented to our hospital. Chest computed tomography (CT) revealed new consolidations of the left inferior lobe despite administration of a moderate dose of oral steroids. Repeated transbronchial lung biopsy showed pulmonary cryptococcosis. The left interior consolidations shrank gradually after antifungal therapy was initiated. Immunocompromised patients with pulmonary cryptococcosis show various CT findings, and consolidation is frequently observed. Superimposed pulmonary cryptococcosis infection should be considered in cases of steroid‐refractory COP.

## Graphical abstract

Immunocompromised patients with pulmonary cryptococcosis show various computed tomography (CT) findings, and consolidation is frequently observed. Superimposed pulmonary cryptococcosis infection should be considered in cases of steroid‐refractory cryptogenic organizing pneumonia (COP).

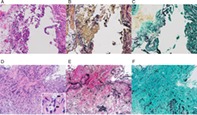

## Introduction

Cryptococcosis is a common fungal infection caused by *Cryptococcus neoformans*, an encapsulated round budding yeast found worldwide in soils contaminated with pigeon droppings, rotten wood, and other organic materials. Humans become infected by inhaling cryptococcal particles, and they develop pneumonia as the initial manifestation of infection; this is followed by granuloma formation. Pulmonary cryptococcosis develops in both immunocompetent and immunocompromised hosts.

Organizing pneumonia (OP) is believed to be an inflammatory repair process triggered after alveolar epithelial injuries. Secondary OP can be associated with a specific cause, such as infection, connective tissue disease, various drugs, radiation, malignancy, and other interstitial pneumonias. If a cause is not identified, the OP is defined as cryptogenic OP (COP). The typical manifestations of COP on computed tomography (CT) are bilateral, patchy, or diffuse consolidations or ground‐glass opacities 1.

COP usually improves considerably with steroid therapy, but relapse can occur when the steroid dose is tapered and stopped. Lazor et al. have reported that one or more relapses occurred in 58% of patients with biopsy‐proven COP, 68% of whom experienced the first relapse during initial steroid therapy. Most patients had been receiving less than 10 mg/day of prednisolone (PSL) at the time of relapse, and relapse rarely occurred in patients receiving a high dose of PSL (>20 mg/day) 2. Some infectious diseases frequently show atypical clinical and radiological manifestations with steroid therapy. Therefore, refractory relapses of COP must be carefully re‐evaluated, mainly when relapse occurs at a high dose of PSL. We report a case involving an immunocompetent patient diagnosed with COP by bronchoscopy who became refractory to a moderate dose of steroids. He was finally diagnosed with pulmonary cryptococcosis by re‐examination with bronchoscopy.

## Case Report

**Figure 1:**
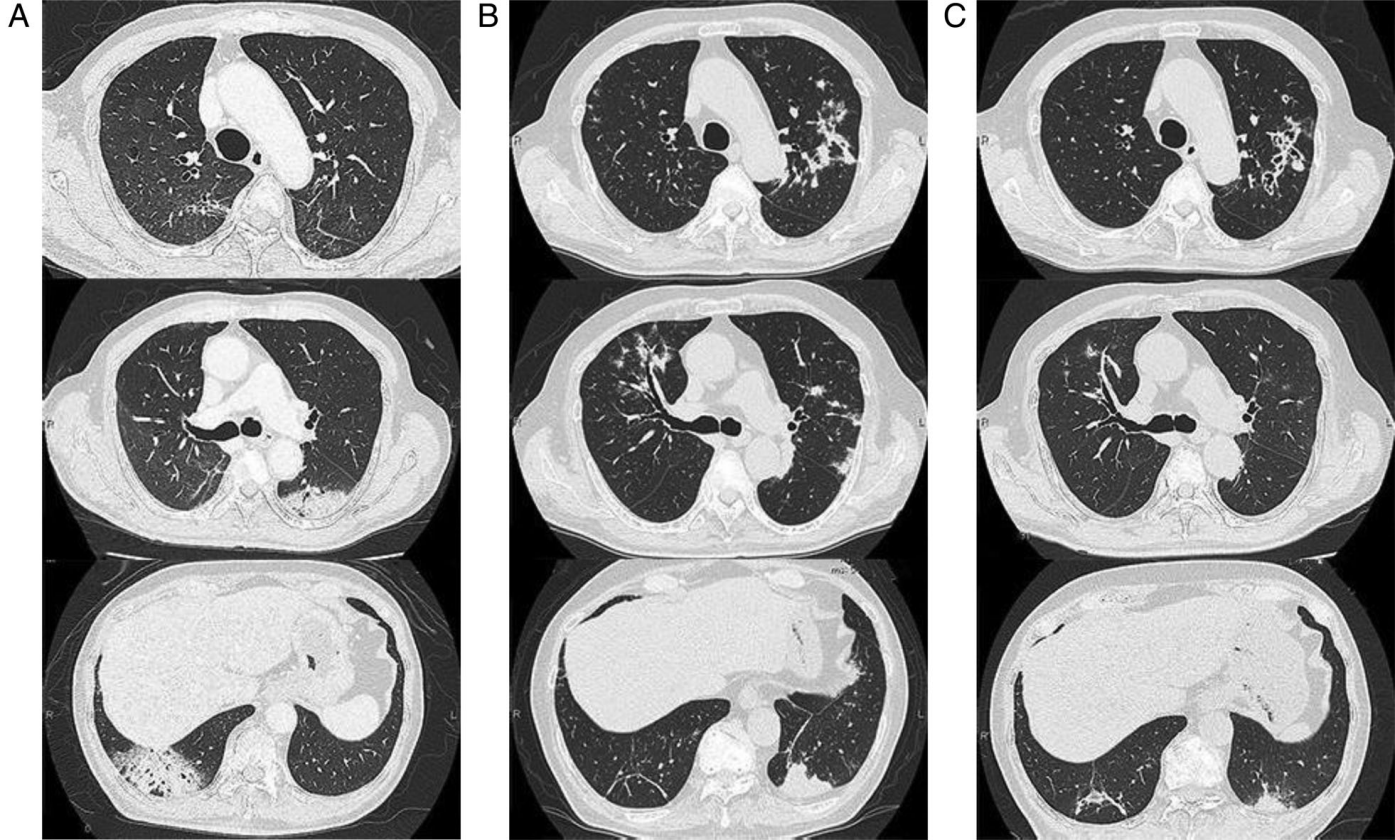
Radiological findings. (A) Chest computed tomography (CT) scan obtained at the time of the initial diagnosis of cryptogenic organizing pneumonia (COP). The corticosteroid had not yet been administered. Consolidations were observed on multiple lobes of the lung. No nodular opacities were observed in the lung. (B) Chest CT scan obtained at the first visit to our hospital showing consolidation of the left lower lobe and multiple nodular shadows with thickened bronchial walls in the lungs bilaterally. Initial consolidations observed at the time of the diagnosis of COP had disappeared. (C) Chest CT scan obtained after taking fluconazole for six months. Consolidations of the left lower lobe had shrunk, and multiple nodular shadows had reduced. Bronchiectasis is observed in the left upper lobe.

**Figure 2:**
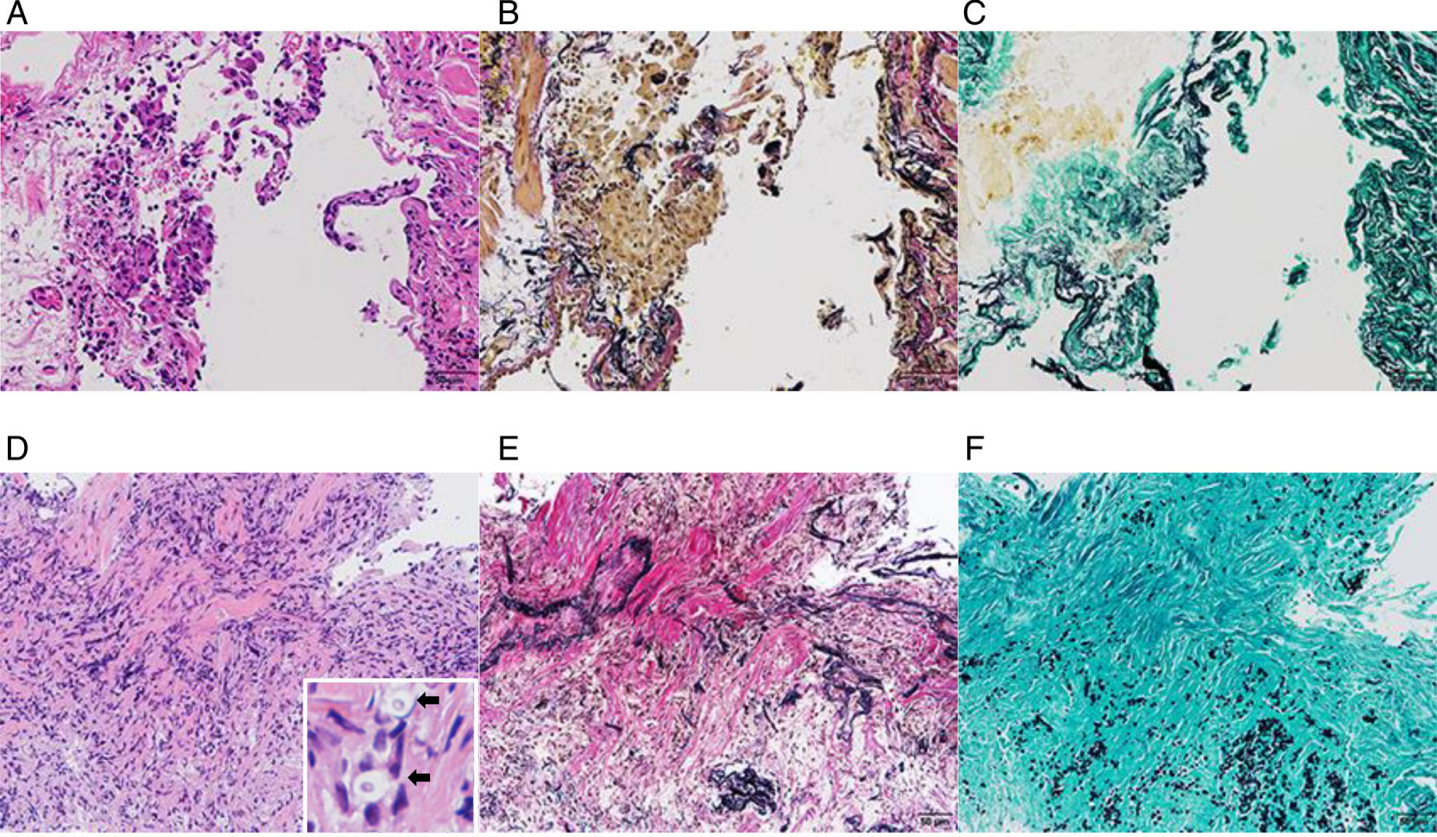
Transbronchial biopsy specimens. (A) Histopathological examination of the transbronchial biopsy specimens taken from the right lower lobe (performed at the previous hospital before corticosteroid therapy was initiated; haematoxylin and eosin staining, 20×). (B) Elastica van Gieson (EVG) staining of the same specimen shown in (A) revealing accumulation of alveolar macrophages and a few fibroblasts in the alveolar spaces near the bronchiole. (C) Grocott staining of the same specimen shown in (A). No yeast or bacterial cells are noted in the tissue. (D) Histopathological examination of the transbronchial biopsy specimens taken from the left lower lobe performed at our hospital (haematoxylin and eosin staining, 10×). (E) EVG staining of the same specimen shown in (D). Multiple yeasts and macrophages are observed in the interstitium and alveolar spaces. Note the small round spheres found in the cytoplasm of macrophages (arrows in the enlarged view in the square). (F) Grocott staining of the same specimen shown in (D) revealing multiple yeasts in the interstitium and alveolar space.

A 74‐year‐old man presented to a local clinic with a 2‐month history of chest discomfort and dyspnoea. His symptoms had not improved with antibiotics. His chest radiography and CT scan showed multiple consolidations in the bilateral lung fields (Fig. 1A). Bronchoalveolar lavage fluid (BALF) analysis showed an elevated lymphocyte differential count (34%), and no microorganisms were cultured from the BALF. Transbronchial lung biopsy (TBLB) specimen taken from the right lower lobe revealed preservation of the underlying architecture and accumulation of alveolar macrophages and a few fibroblasts in alveolar spaces near the bronchiole, although intraluminal organizing fibrosis within alveoli and bronchioles was not identified (Fig. 2A, B). Gram staining, acid‐fast staining, and Grocott staining of the lung tissue were negative (Fig. 2C). Considering these findings, the patient was diagnosed with COP and was administered oral corticosteroid therapy, PSL, 40 mg/day (0.75 mg/kg/day). One month later, his symptoms and chest radiography findings had improved. The corticosteroid dose was then gradually tapered, and when the dose of PSL was reduced to 10 mg/day, chest radiography and CT findings showed exacerbation of the bilateral consolidation. After the PSL dose was increased to 40 mg/day, majority of the consolidations improved again. However, when the dose was decreased to 15 mg/day, chest radiography and CT showed new consolidations on the left inferior lobe. The PSL dose was increased to 40 mg/day once again, and he was referred to our hospital.

The patient's vital signs were normal on his first visit to our hospital, with no abnormalities noted upon physical examination except peripheral numbness in both feet. He had been treated for spinal stenosis, hypertension, and hyperuricaemia but was otherwise healthy. He worked as a cucumber farmer and had constant exposure to fertilizer made from chicken droppings. Laboratory results showed a normal blood cell count and biochemical test results. Chest radiography showed infiltrative shadows in the left lung field, and chest CT revealed consolidation of the left lower lobe and multiple bilateral patchy opacities with thickened bronchial walls (Fig. 1B). Bronchoscopy was performed again and the BALF from the left lingular lobe showed elevation of lymphocyte cells (20.1%). Histopathological examination of the TBLB from the left lower lobe revealed a mass of yeast and infiltration of macrophages in the alveolar space. Some round yeasts were phagocytized by macrophages (Fig. 2D–F). The BALF culture was negative. His serum cryptococcal antigen titre was positive, with a titre of 1:2048, and his serum β‐D glucan tested negative. Although lumbar puncture specimens could not be obtained because of his severe lumbar curvature disease, contrast‐enhanced brain magnetic resonance imaging did not show any signs of meningitis, and he showed no clinical symptom of meningitis. Thus, the patient was diagnosed with pulmonary cryptococcosis without meningitis. He was started on fluconazole 400 mg/day, and PSL dose was gradually decreased. After six months, his PSL dose had been reduced to 5 mg/day, and the consolidations of the left lung had steadily shrunk, as noted on his chest CT. Most of the nodular shadows had disappeared, resulting in dilated bronchial tubes (Fig. 1C).

## Discussion

The most common radiological findings of pulmonary cryptococcosis in an immunocompetent patient are nodules and masses with or without cavitation 3, 4. Most cases that present with consolidations also have nodules or cavities. In immunocompetent patients, Cryptococcus activates the Th1 response. Lymphocytes and macrophages surround the Cryptococcus and form a granuloma, which is shown as nodules on chest CT. Murayama et al. analysed high‐resolution CT findings of 13 immunocompetent patients with pulmonary cryptococcosis 3. They found that the main manifestations were classified into two patterns: multiple nodules (*n* = 7) and a single nodule (*n* = 6). A consolidation pattern with multiple nodules was observed in two cases.

Immunocompromised patients with pulmonary cryptococcosis reveal various CT findings. In these patients, the Th1 response is not activated enough to form a granuloma and fungi tend to diffuse to the surrounding tissues. The frequency of consolidation is higher in immunocompromised patients than in immunocompetent patients 5, 6. Yanagawa et al. compared CT findings of patients with pulmonary cryptococcosis who were immunocompetent, had AIDS and rheumatoid arthritis (RA), and used PSL 5. Nodules were the most common findings in all groups. Consolidation and ground‐glass attenuation were found in 30% of the AIDS and RA groups, but they were not found in the immunocompetent group.

Several case reports of pulmonary cryptococcosis have revealed radiological and pathological findings of OP (Table 1) 7, 8, 9, 10, 11, 12, 13, 14. Most of those patients were immunocompromised and presented bilateral consolidation on chest CT; however, two immunocompetent patients presented consolidations on chest CT. Three cases were refractory to steroids or immunosuppressants. All cases were successfully treated with an antifungal drug.

**Table 1:** Comparison among cases of pulmonary cryptococcus showing OP.

Case	Author (year)	Age/sex	Background disease	Chest radiological findings	Pathological findings	Serum cryptococcal antigens	Therapy	Outcome
1	Kishi (2004) 9	31/M	None	Consolidations and patchy opacities	OP pattern	+	Fluconazole	Improved
2	Ouchi (2005) 12	54/M	DM	Bilateral consolidations	Cryptococcus phagocytosed by macrophages	+	Fluconazole/itoraconazole	Improved
3	Chantranuwat (2005) 8	67/M	DM	Bilateral consolidations, nodules (CT)	OP pattern, Cryptococcus in alveolar macrophages	N/A	Amphotericin B/fluconozole	Improved
4	Taniguchi (2010) 13	78/M	DM	Bilateral consolidations and patchy opacities	Necrosis, granuloma, multinucleated giant cell with Cryptococcus	+	Fluconazole	Improved
5	Kessler (2010) 11	30/M	None	Bilateral consolidations, nodules (CT)	OP pattern, multinucleated giant cell with Cryptococcus	−	Fluconazole	Improved
6	Katsurada (2012) 14	68/F	SjS, (administration of PSL)	Bilateral consolidations	Cryptococcus in alveolar histiocytes	+	Fluconazole	Improved
7	Chikumoto (2019) 10	65/F	Neurosarcoidosis (administration of PSL, MTX)	Bilateral non‐segmental consolidations, multiple nodules	OP pattern, Cryptococcus	+	Fluconazole	Improved
8	Chikumoto (2019) 10	72/M	RA (administration of PSL, MTX, anti‐TNF‐α)	Bilateral non‐segmental consolidations, multiple nodules	N/A	+	Fluconazole	Improved

CT, computed tomography; DM, diabetes mellitus; MTX, methotrexate; N/A, not applicable; OP, organizing pneumonia; PSL, prednisolone; RA, rheumatoid arthritis; Sjs, Sjogren's syndrome; TNF‐α, tumour necrosis factor‐alpha.

There are two case reports of patients who were initially diagnosed with COP and re‐diagnosed with pulmonary cryptococcosis during steroid therapy (Table 2) 14, 15. In one case, Katsurada et al. concluded that pulmonary cryptococcosis was misdiagnosed as COP in a patient owing to the absence of pathological examination 14. In the other case, Tashiro et al. considered that the patient with COP had an opportunistic Cryptococcus infection after the administration of steroid treatment because the first pathological and bacterial examination by bronchoscopy showed no evidence of cryptococcal infection, and the initial consolidation improved only with steroid therapy 15.

**Table 2:** Comparison of two patients whose diagnosis had been changed from COP to pulmonary cryptococcosis during steroid therapy and the patient in our case.

case	Author (year)	Age/sex	Background disease	CT findings at the “initial” diagnosis	The initial” diagnosis	Reason for the initial diagnosis	CT findings at the relapse	The “secondary” diagnosis	Reason for the secondary diagnosis
1	Tashiro (2003) 15	65/M	None	Bilateral consolidations	COP	No pathogenic microorganism in BALF	Bilateral consolidations, nodules	Pulmonary cryptococcosis	Positive for serum antigen Yeast‐like fungi in BALF
2	Katsurada (2012) 14	68/F	SjS, (administration of PSL)	Bilateral consolidations	COP	Only radiological findings	Bilateral patchy consolidations	Pulmonary cryptococcosis	Positive for serum antigen Yeast‐like fungi in BALF and lung specimen
3	Nomura (2020) [this study]	74/M	None	Bilateral consolidations	COP	No pathogenic microorganism in BALF	Bilateral consolidations, nodules	Pulmonary cryptococcosis	Positive for serum antigen
						OP pattern in the lung specimen			Yeast‐like fungi in BALF and lung specimen

BALF, bronchoalveolar lavage fluid; COP, cryptogenic organizing pneumonia; CT, computed tomography; OP, organizing pneumonia; PSL, prednisolone; Sjs, Sjogren's syndrome.

It is essential to discriminate COP from secondary OP because the management of secondary OP often needs treatment of the underlying disease; however, it is not easy to distinguish secondary OP from COP in clinical practice. Drakopanagiotakis et al. recently reported that the clinical and radiological findings in patients with COP and secondary OP are similar and non‐specific 16.

Serum antigen testing for cryptococcosis may be useful to differentiate pulmonary cryptococcosis and COP. The sensitivity and specificity of serum antigen tests were examined using 195 sera from 25 patients with pulmonary cryptococcosis and 170 patients with non‐cryptococcosis. A cut‐off value of ≥1:8 showed a sensitivity of 76% (19/25) and specificity of 98.9% (168/170) 17. In our review, the serum antigen test for cryptococcosis was examined in seven patients among those with pulmonary cryptococcosis who showed an OP pattern on chest CT, and six of them tested positive (Table 2).

We suspected three main reasons as to why our patient with COP developed secondary pulmonary cryptococcosis. First, the initial radiological finding of this patient was multiple consolidations without nodules, which is rare on the chest CT scan of an immunocompetent patient with pulmonary cryptococcosis; second, the consolidations on chest CT scan at the time of diagnosis of COP were sensitive to the steroids; third, cryptococcosis yeast was not detected in the result of the first bronchoscopy before the administration of PSL.

The patient used fertilizer made from chicken droppings at his farm every day. Therefore, he was exposed to Cryptococcus on a daily basis, and not surprisingly, he was infected by Cryptococcus during steroid therapy. However, there is a possibility that he had been infected by Cryptococcus at the time of his first symptom; serum cryptococcal antigen was not examined before the corticosteroid was administrated.

In conclusion, pulmonary cryptococcosis can result in multiple consolidations on chest CT in immunocompetent and immunocompromised patients. It is rare, but a superimposed infection of pulmonary cryptococcosis should be considered in cases of COP non‐responsive to steroids, especially when deterioration is observed after an initial improvement through steroid therapy.

### Disclosure Statement

Appropriate written informed consent was obtained for publication of this case report and accompanying images.
